# Who does singlehood best? A latent profile analysis

**DOI:** 10.3389/fpsyg.2025.1509349

**Published:** 2025-07-21

**Authors:** Calen J. Horton, Lisa C. Walsh, Anthony Rodriguez, Victor A. Kaufman

**Affiliations:** ^1^Arkoda Research Group, Anchorage, AK, United States; ^2^Division of Psychology, School of Social Sciences, Nanyang Technological University, Singapore, Singapore; ^3^RAND Corporation, Santa Monica, CA, United States; ^4^Department of Psychology, University of California, Los Angeles, Los Angeles, CA, United States

**Keywords:** singlehood, dating, pressure to partner, well-being, ill-being, personality, socialization

## Abstract

Researchers have begun to address heterogeneity in the single population. Historically, singles have been differentiated either by their marital history (e.g., widowed, divorced, never-married) or by whether they considered their singlehood voluntary. However, these approaches leave many unanswered questions about heterogeneity among singles. Addressing heterogeneity is important in light of recent interest in understanding how well-being varies across categories of singles. In the present study, we address this question by using Latent Profile Analysis (LPA) on a large cross-sectional sample of American singles (*N* = 4,835). Using LPA, we identified nine distinct profiles of singles differentiated by their romantic relationship goals (partner-seekers, casual-daters, and non-daters) and the perceived pressure they report experiencing from others to find a romantic partner (low-pressure, medium-pressure, and high-pressure). We then compared the profiles on levels of well-being, ill-being, personality, and socialization, as well as demographic, socioeconomic, and relationship history variables. Our analysis reveals several patterns of cross-category variability in outcomes, including some outcomes that vary primarily by romantic relationship goals, some that vary primarily by pressure, and others that vary according to a combination of both. Our results also suggest that two groups (low-pressure casual-daters and low-pressure non-daters) fare particularly well. They are also relatively common, comprising 30% of the sample. A third group—high-pressure non-daters—fare very poorly, and though they comprise only a small proportion of the sample (1.5%) the implications of this group are substantial when considered at national scale. The implications of these findings for research on singlehood and well-being are discussed.

## 1 Introduction

Who does singlehood best? This question is a focal point of the current wave of singlehood research. Our review of singlehood research suggests the field has seen three waves.

The first wave was characterized by general neglect—singles were largely used as a comparison group to study marriage. Some researchers did study singles as a unique group (e.g., Spreitzer and Riley, 1974; Stein, 1975, 1978), but the attitude towards singles was relatively dismissive. Generally, unhappy singles were used as a counterpoint to promote marriage as the key to a happy life (DePaulo and Morris, 2005). Despite cultural beliefs that inculcated these attitudes, singlehood in the U. S. grew from 28% in 1960 to 49% in 2020, with an especially noteworthy rise among younger adults (Marino, 2023). This trend owes much to a decrease in partnering rather than an increase in unmarried cohabitation: between 1990 and 2019, the share of unpartnered singles in the U. S. rose from 29 to 38% (Fry and Parker, 2021).

The second wave of singlehood research emerged from this demographic shift. The inflection point was an article by DePaulo and Morris (2005) addressing scientific and cultural biases against singles. The driving force of the second wave was skepticism toward marriage-centric treatments of singlehood and their accompanying biases (Morris et al., 2008). Researchers also challenged the methodology of previous research; as DePaulo (2017) noted, many older cross-sectional studies overstated the benefits of marriage by excluding those who divorced—or worse, lumping them together with lifelong singles. Research during this time also documented anti-single bias across various domains, including government policies, interpersonal discrimination, and negatively biased evaluations (Byrne and Carr, 2005; DePaulo and Morris, 2005).

The third and current wave sprung from research conducted during the second wave. It is characterized by two main features. The first feature emphasizes well-being. Since earlier research primarily emphasized couplehood, factors promoting well-being in singles were largely ignored. Therefore, third-wave singlehood researchers have devoted substantial resources toward determining factors that predict well-being and ill-being in singles, both as a unique population (see Kislev, 2018, 2022; Girme et al., 2016, 2022; Walsh et al., 2022) and in comparison to couples (Walsh et al., 2023). The second feature emphasizes heterogeneity. The label “single” encompasses a wide range of experiences, from “mama’s boys” to “cat ladies,” celibates to divorcees, widows to never-marrieds, and single parents of all varieties (Dupuis and Girme, 2024). Third-wave researchers have sought to categorize singles’ individual experiences (Kislev, 2023) and map how well-being varies across demographics such as gender, race, and age (Kislev and Marsh, 2023).

A major goal of the third wave of singlehood research has been to move beyond deficit-based narratives of singlehood—narratives that are predicated on the assumption that humans are only “complete” when coupled, and that singles are therefore either morally or psychologically diminished by the state of singlehood (DePaulo, 2023a). In the beginning stages of the third wave, researchers approached this in different ways. DePaulo focused on the singles-studies perspective, which emphasized that many single individuals are flourishing, leading psychologically rich lives characterized by freedom, autonomy, and deep connections with multiple people rather than centering their lives around a single person (i.e., a romantic partner). Others, however, have taken a different approach. For example, Girme et al. (2023) expressed concern about framing certain singlehood experiences as deficit narratives. Instead, they emphasized the role of “secondary control”—a psychological process in which individuals adapt to unchangeable circumstances by reframing their understanding of their situation (Hostetler, 2009). In the context of singlehood, this applies to widows, divorcees, solo parents, and marginalized individuals who may be involuntarily single or face challenges in finding romantic opportunities. Girme et al. (2023) argued that the processes of growth, change, and adjustment in these situations should not be dismissed as deficit-based but rather recognized alongside more stable narratives of choosing singlehood.

One of the current challenges facing singlehood research—and reconciling these different viewpoints—is finding a way to integrate this emerging conceptualization of singlehood within a holistic framework that captures the full diversity of single experiences. While many singles naturally defy deficit narratives, living fulfilling and autonomous lives, others undergo a process of reflection and reappraisal to reach a similar sense of well-being—a process that aligns with secondary control (Girme et al., 2023; Hostetler, 2009). Some individuals, for example, become long-term singles due to the loss of a partner or other life circumstances. While they may not initially welcome their single status, their journey towards finding contentment in singlehood is an important focus for researchers. As Kislev (2023) noted, many singles view their singlehood as a temporary state—planning to seek a partner and settle down when the opportunity arises.

An open question, then, is how to develop a generous science of singlehood—one that acknowledges and embraces the full spectrum of individual experiences while pushing back against rhetoric that portrays singles as an unhappy and undifferentiated monolith. Notably, marriage research allows for variability and studies it frequently—it has been recognized for decades, for example, that there is a category of married people who experience long-term unhappy but stable marriages (see Heaton and Albrecht, 1991). The challenge for singlehood research, then, is to establish a similar degree of nuance, ensuring that the complexities of single life are understood and represented with the same depth and sophistication.

To that end, we note that a pressing and unresolved issue in establishing such nuance is that we simply do not have a clear understanding of the different types of singles, their prevalence, and the psychological characteristics that define their singlehood. In a recent exchange between DePaulo (2023b) and Girme et al. (2023), DePaulo summed up this challenge to third-wave singlehood research elegantly: Who does the single life best? Or, asked a different way, what are the different “types” of singles, how happy are they, and what factors account for happiness differences among them? The present study aims to find out.

### 1.1 Heterogeneity in singlehood

The first step in addressing the question of who does the single life best is to divide singles into meaningful categories. Indeed, some people experience singlehood as a trap to be escaped, others experience it as a game to be played, and still others experience it as a happy alternative to be embraced. A reasonable taxonomy of singles should address this internal variation in the lived experience of singlehood.

For decades this question was left mostly untouched. Until recently, the primary method of classifying singles was to divide them into widowed, divorced, separated, and never-married categories (e.g., Spreitzer and Riley, 1974). Stein (1978) pioneered an alternative approach, classifying singlehood on the dimensions of voluntariness and stability—laudably differentiating singles based on their own evaluations rather than the presence or absence of a prior marriage. However, as Kislev (2023) noted, both approaches still overlook singles’ lived experiences. Instead, they address external factors such as how one’s singlehood started, whether it was preceded by marriage, and its anticipated duration.

Recent treatments are more relevant to the lived experience of singles, with three developments worth noting. First, Pew Research has issued multiple reports categorizing singles based on relationship intentions—that is, whether they are seeking a long-term partner, casual dates, either, or not looking at all (Brown, 2020, 2022). These reports depart from older treatments of singlehood since they taxonomize singles based on internal factors (e.g., romantic goals) rather than external categories (e.g., marital status).

Second, Kislev (2023) has argued that singlehood should be considered an identity, segmenting singles’ identities into three broad “types”: (1) a core identity, where one’s singlehood is a positive, stable source of self-definition; (2) a peripheral identity, where one views singlehood as temporary and actively flirts with relationships via casual dating; and (3) a counter-normative identity, where singlehood is experienced as a stable but stigmatized identity, accompanied by feelings of exclusion. Kislev and Marsh (2023) further argued that singlehood researchers should take an intersectional approach to singlehood, considering how single identity combines with other categories such as race, gender, and sexuality. While this approach is promising, thus far, no instruments have been created to assess singlehood as an identity, and the groupings proposed by Kislev (2023) remain theoretical.

A final key development is the growing use of methods that combine algorithmic, rule-based data partitioning (e.g., latent profile analysis [LPA]) with strong theoretical guidance to produce empirically grounded taxonomies of singles. Although LPA is often described as a data-driven strategy (e.g., Bauer, 2022; Wardenaar, 2021), it is essential that the resulting groups maintain clear theoretical coherence (Nylund-Gibson and Choi, 2018; Masyn, 2013). Notably, it is the researcher’s responsibility to ensure the resulting groups are also of theoretical importance. Researchers have started applying this approach to better understand singlehood. For example, Park et al. (2023) used LPA to typologize singles based on the Fundamental Social Motives Inventory (Neel et al., 2016), which measures affiliative, disease avoidant, mate-seeking, and self-protection motives. Similarly, Walsh et al. (2022) used LPA to provide a ten-profile typology of singles based on predictors of life satisfaction in the domain of personal relationships, self-esteem, and personality. Finally, Pepping et al. (2024) used LPA to create a typology of singles based on attachment styles, finding a four-profile solution that mirrors the four primary attachment styles.

LPA-based approaches offer a strong basis for typology because they are empirically grounded. The sole caveat is that LPA results depend on the variables selected as indicators to identify latent profiles; the chosen indicators act as a “lens” that allows the researcher to split a sample into subgroups according to a topic of interest. Thus, Park et al. (2023) view divisions among singles through the lens of fundamental social motives, Walsh et al. (2022) view them through the lens of well-being, and Pepping et al. (2024) view them through the lens of attachment.

Each “lens” provides valuable insight. One lens which, to our knowledge, has not been used to examine divisions between singles is their self-reported experience—that is, their romantic goals and desires, and their perceptions of others’ reactions to their singlehood. This overlaps closely with Kislev’s (2023) approach to studying singlehood as an identity, and may be a highly informative and natural approach to uncover “types” of singles.

### 1.2 Research questions

Accordingly, the present study uses LPA to address three research questions. First, can singles be fruitfully classified into “types” based on their own self-reported experience—and, if so, what are the primary factors determining their “type”?

Second, who does singlehood best? That is, given a battery of measures related to well-being and ill-being, personality (e.g., extraversion, self-esteem) and socialization (e.g., friend and family satisfaction), are there “types” of singles who fare notably better or worse? Are there groups that experience more complex combinations of well-being and ill-being? Research suggests that well-being and ill-being are sometimes separable (e.g., Horton et al., 2024), so some types of singles may exhibit higher (or lower) levels of both well-being and ill-being.

Third, do different “types” of singles map onto the population in recognizable ways? Colloquially, laypeople differentiate between types of singles (e.g., “cat ladies,” “mama’s boys”; Dupuis and Girme, 2024). An open question is whether a taxonomy of singles might produce groups that are demographic analogues of recognizable lay-categories—and, if so, how do those demographic groupings relate to well-being? For example, there may be demographic trends across groups that mirror findings in previous studies, such as the tendency of older people to be happy (Frijters and Beatton, 2012) or the tendency of men to engage in more casual sex (England and Bearak, 2014).

### 1.3 Why latent profile analysis?

Latent Profile Analysis (LPA) is a person-centric analytic method that divides a sample into heterogeneous groups (referred to as profiles) based on variations in the levels of multiple input variables (referred to as indicators). It contrasts with variable-centric analyses such as regression, which summarize relationships between variables (Ferguson et al., 2020; Spurk et al., 2020; Woo et al., 2018).

LPA is well-suited to addressing the question of singlehood “types” for two reasons. The first reason is that LPA can parsimoniously represent complex, multi-variable data structures by instantiating them as groups (Woo et al., 2018), whereas variable-centric methods such as regression add multiple interaction terms for every new variable to account for the same level of complexity.

The second reason is that variable-centric methods presume a linear relationship between variables, while LPA handles complex non-linear relationships easily. LPA researchers distinguish two patterns of difference across profiles. The first type, level differences, describes a linear relationship between indicators, such that indicators rise (or fall) in tandem with each other across profiles. The second type, shape differences, indicates a relationship where indicators vary independently, forming idiosyncratic combinations across profiles. Shape differences are prized among LPA researchers, as they indicate patterns that might otherwise have been missed by variable-centered methods.

### 1.4 The present study

We use LPA to characterize singlehood using intrinsic and extrinsic indicators, grounded in motivational science (Ryan and Deci, 2000) and foundational theories (Lewin, 1939). Intrinsic factors reflect internal desires or interests. Our intrinsic indicators for singles include the type of relationship wanted (e.g., committed partner, casual dates; i.e., romantic goals), desire to find a romantic partner, and dating frequency. Extrinsic factors stem from external forces, real or perceived. Cialdini et al. (1991) categorized external factors into injunctive norms (perceptions of socially approved or disapproved behaviors) and descriptive norms (perceptions of what others actually do). Accordingly, our extrinsic indicators for singles include injunctive norms (perceived social pressure to partner) and descriptive norms (the presence of married/partnered people within one’s social network). Each indicator is described further in the Measures section below.

Using these intrinsic and extrinsic indicator variables, we aim to develop a typology of singlehood and examine how it maps onto important real-world psychological factors (well-being, ill-being, personality, socialization), demographic characteristics (e.g., age, gender, socioeconomic status), and diverse relationship histories (e.g., monogamous, casual). We expect this approach to provide a more nuanced understanding of singles’ lived experiences, illuminating how internal desires and external pressures jointly shape singlehood.

## 2 Materials and methods

### 2.1 Participants and procedures

The present study is an exploratory analysis of a dataset collected in 2021 via Dynata. For prior studies using the same dataset, (see Walsh et al., 2022, 2023). The initial participant pool was filtered according to age and relationship status; prospective participants were allowed to take the survey if they indicated they were single and between 18 and 65 years old. Except for these two criteria, participants were otherwise matched to U.S., census targets. Participants were provided with a link to an online survey and were compensated with cash, reward points, or discounts. All procedures were reviewed by the local institutional review board.

To ensure data quality, seven engagement checks were embedded into the survey to ensure participants were paying attention. Participants who failed any engagement checks were excluded, as were participants who straight-lined (selecting the same answer repeatedly) through four or more scales. This reduced the final sample size from 5,010 to 4,835 single adults. See Tables 1 and 2 for the full sample (*N* = 4,835) demographics.

**Table 1:** Profile demographics—gender, age and race/ethnicity.

Profile designation	Full sample	Partner-seekers			Casual-daters			Non-daters		
		Low pressure	Medium pressure	High pressure	Low pressure	Medium pressure	High pressure	Low pressure	Medium pressure	High pressure
Profile name		Relaxed seekers	Lonesome seekers	Complex seekers	Happy explorers	High social explorers	Stressed explorers	Happy non-daters	Introverted non-daters	Distressed non-daters
N (%)	4,835 (100%)	960 (19.9%)	871 (18.0%)	382 (7.9%)	402 (8.3%)	434 (9.0%)	340 (7.0%)	1,064 (22.0%)	309 (6.4%)	73 (1.5%)
Gender										
Female	57.5% (0.8)	49.4% (1.9) ^3^	60.7% (1.9) ^2^	64.2% (2.7) ^1^	43.0% (2.7) ^3^	46.8% (3.1) ^3^	44.8% (3.3) ^3^	67.6% (1.5) ^1^	71.2% (2.9) ^1^	69.8% (5.8) ^1^
Male	42.5% (0.8)	50.6% (1.9) ^1^	39.3% (1.9) ^2^	35.8% (2.7) ^3^	57.0% (2.7) ^1^	53.2% (3.1) ^1^	55.2% (3.3) ^1^	32.4% (1.5) ^3^	28.8% (2.9) ^3^	30.2% (5.8) ^3^
Age										
18–24	22.2% (0.6)	14.9% (1.1)	35.2% (1.6)	31.7% (2.4)	14.8% (1.8)	33.1% (2.3)	27.6% (2.4)	6.6% (0.8)	29.4% (2.6)	42.0% (5.8)
25–44	32.6% (0.7)	29.5% (1.5)	39.2% (1.7)	50.1% (2.6)	19.7% (2.0)	38.2% (2.3)	62.4% (2.6)	15.9% (1.1)	33.4% (2.7)	35.8% (5.6)
45–65	45.2% (0.7)	55.6% (1.6)	25.6% (1.5)	18.1% (2.0)	65.4% (2.4)	28.7% (2.2)	10.0% (1.6)	77.6% (1.3)	37.2% (2.7)	22.1% (4.9)
Race/Ethnicity										
Asian	6.6% (0.4)	4.1% (0.8)	9.7% (1.1)	7.4% (1.5)	8.5% (1.5)	8.6% (1.7)	6.8% (1.7)	3.4% (0.6)	8.6% (1.8)	6.7% (3.2)
Black/African American	14.5% (0.5)	13.7% (1.3)	16.1% (1.5)	16.2% (2)	12.4% (1.8)	17.1% (2.4)	23.2% (2.8)	9.1% (0.9)	19.6% (2.5)	10.3% (4)
Hispanic/Latinx	13.8% (0.5)	11.8% (1.2)	15.1% (1.4)	22.5% (2.3)	10.4% (1.7)	20.1% (2.4)	18.2% (2.6)	6.8% (0.8)	15.7% (2.3)	25.4% (5.5)
White	71.1% (0.7)	76.2% (1.6)	67.6% (1.9)	61.4% (2.7)	74.3% (2.4)	63.0% (3)	58.6% (3.3)	82.1% (1.2)	63.8% (3.1)	65.9% (6)

Cells presented as %(SE) or M(SE). For Race/Ethnicity, participants were able to select more than one category so percentages may not sum to 100%. Standard Errors for Age estimated using the formula SE = $\sqrt{p*(1-p)/n}.$ Percentages and standard errors for Gender and Race/Ethnicity were taken from DCAT results. Superscripts 1–3 indicate tiers: 1 = the highest tier consisting of the highest-scoring profile on a given variable and any other profiles that were not significantly different from it. 3 = the lowest tier consisting of the lowest-scoring profile and any other profiles that were not significantly different from it. 2 = the middle tier consisting of profiles that did not fit into either the highest or lowest tiers.

**Table 2:** Profile demographics – socioeconomic variables and singlehood type.

Profile designation	Full sample	Partner-seekers			Casual-daters			Non-daters		
		Low pressure	Medium pressure	High pressure	Low pressure	Medium pressure	High pressure	Low pressure	Medium pressure	High pressure
Profile name		Relaxed seekers	Lonesome seekers	Complex seekers	Happy explorers	High social explorers	Stressed explorers	Happy non-daters	Introverted non-daters	Distressed non-daters
N (%)	4,835 (100%)	960 (19.9%)	871 (18.0%)	382 (7.9%)	402 (8.3%)	434 (9.0%)	340 (7.0%)	1,064 (22.0%)	309 (6.4%)	73 (1.5%)
Income										
Below $50 k	49.8% (0.7)	51.9% (1.6)	47.0% (1.7)	45.0% (2.6)	52.2% (2.5)	40.5% (2.4)	27.6% (2.4)	61.6% (1.5)	47.7% (2.8)	59.8% (5.7)
$50 K to $100 k	37.4% (0.7)	35.3% (1.5)	39.3% (1.7)	37.6% (2.5)	37.0% (2.4)	41.9% (2.4)	54.3% (2.7)	30.1% (1.4)	42.1% (2.8)	29.2% (5.3)
Over $100 k	12.8% (0.5)	12.8% (1.1)	13.8% (1.2)	17.6% (2.0)	10.8% (1.6)	17.5% (1.9)	18.2% (2.1)	8.2% (0.8)	10.2% (1.7)	11.0% (3.6)
Education (Highest reached)										
High school	22.1% (0.6)	22.5% (1.3)	21.0% (1.4)	20.9% (2.1)	20.6% (2.0)	17.1% (1.8)	20.2% (2.2)	25.1% (1.3)	24.8% (2.5)	27.6% (5.2)
College	64.8% (0.7)	66.2% (1.5)	67.0% (1.6)	63.3% (2.5)	66.7% (2.3)	66.4% (2.3)	64.7% (2.6)	61.2% (1.5)	63.6% (2.7)	64.4% (5.6)
Postgraduate	13.0% (0.5)	11.2% (1.0)	11.9% (1.1)	15.7% (1.9)	12.7% (1.7)	16.5% (1.8)	14.9% (1.9)	13.5% (1.0)	10.9% (1.8)	8.0% (3.2)
Prior marital status										
Widowed	5.9% (0.3)	4.9% (0.7)	1.6% (0.4)	2.6% (0.8)	8.7% (1.4)	4.6% (1.0)	2.1% (0.8)	12.8% (1.0)	4.5% (1.2)	5.5% (2.7)
Divorced	19.8% (0.6)	27.9% (1.4)	12.9% (1.1)	9.9% (1.5)	24.9% (2.2)	11.8% (1.6)	9.7% (1.6)	26.8% (1.4)	21.0% (2.3)	8.2% (3.2)
Separated	3.7% (0.3)	3.0% (0.6)	3.7% (0.6)	4.5% (1.1)	2.2% (0.7)	5.5% (1.1)	6.5% (1.3)	3.7% (0.6)	2.3% (0.9)	1.4% (1.4)
Never Married	70.5% (0.7)	64.2% (1.5)	81.9% (1.3)	83.0% (1.9)	64.2% (2.4)	78.1% (2.0)	81.8% (2.1)	56.8% (1.5)	72.2% (2.6)	84.9% (4.2)
Household characteristics										
Live alone	47.9% (0.7)	52.1% (1.6)	43.2% (1.7)	40.3% (2.5)	54.7% (2.5)	46.3% (2.4)	47.4% (2.7)	52.8% (1.5)	37.5% (2.8)	52.1% (5.9)
Attending school	13.9% (0.5)	10.4% (1.0)	21.1% (1.4)	18.8% (2.0)	9.4% (1.5)	21.0% (2.0)	20.6% (2.2)	4.2% (0.6)	18.4% (2.2)	21.9% (4.8)
Child in household	16.3% (0.5)	14.8% (1.1)	15.7% (1.2)	17.5% (1.9)	15.7% (1.8)	18.4% (1.9)	25.0% (2.4)	14.0% (1.1)	17.8% (2.2)	15.1% (4.2)

Cells presented as %(SE) or M(SE). Standard Errors estimated using the formula SE = $\sqrt{p*(1-p)/n}.$

### 2.2 Measures

#### 2.2.1 Indicators for latent profile analysis

In LPA, indicators are the input variables used to identify and define latent profiles within a sample. We used the below intrinsic and extrinsic factors as indicators.

##### 2.2.1.1 Intrinsic factors

Type of relationship desired was assessed using a question adapted from Brown (2020) that asked participants if they were looking for a committed partner, casual dates, or neither. Participants were able to select both the “committed partner” and “casual dates” option, but if they indicated they were not looking for a partner or dates, they were not allowed to choose another option. Because participants could select multiple responses, their answers were coded into three separate variables: (1) committed partner, (2) casual dates, and (3) no dating (1 = *Yes*; 0 = *No*).

Desire to partner was assessed using a scale adapted from Blakemore et al. (2005). Participants responded to six items (e.g., “I cannot wait to have a romantic partner”) using a 4-point scale assessing their agreement (1 = *Strongly disagree*; 4 = *Strongly agree*). The resulting average composite demonstrated good reliability according to Cronbach’s alpha (*α* = 0.86). Scales assessing desire to remain single have not yet been developed, so we used desire to partner as a reverse-coded proxy for singlehood—the logic being a strong desire to couple is generally incompatible with a strong desire to remain single (see Kislev, 2021 for a paper using this approach).

Dating frequency was assessed using a single item, “Before the COVID-19 pandemic, how frequently did you go on dates?” Participants rated their response on a 7-point scale (1 = *Multiple times a week*; 7 = *Never*); this item was reverse-coded such that higher values reflected more frequent dating. Since data were collected during the COVID-19 pandemic, we asked participants about their dating frequency pre-COVID, reasoning it would be a truer reflection of their preferences than a measure affected by the exigent circumstances of the pandemic.

##### 2.2.1.2 Extrinsic factors

Perceived pressure to partner (an injunctive norm) was assessed using three questions adapted from Brown (2020). Participants were asked how much pressure to partner, if any, they felt from their family, friends, and society at large. Participants answered using a 4-point scale (1 = *No pressure at all*; 4 = *A lot of pressure; α* = 0.74).

Married people in network (a descriptive norm) was assessed with a single item, “How many people in your social network are married or are in a committed romantic relationship?” Participants rated their response on a 4-point scale (0 = *No married people in network*; 3 = *3+ married people in network*). Notably, this variable may carry additional meanings beyond normative pressure.

#### 2.2.2 Outcomes and covariates

In LPA, outcomes are dependent variables used to assess the effects of profile membership on behaviors, attitudes, or other constructs. They reveal how belonging to a specific profile relates to various dimensions under study. In contrast, covariates are external variables associated with profile membership but do not shape the profiles themselves. Unlike in regression, LPA covariates are not treated as predictors or controls; instead, they help clarify how explanatory variables correspond to profile membership. We assessed the following outcomes and covariates.

##### 2.2.2.1 Well-being

Life satisfaction was assessed using the five-item Satisfaction With Life Scale (SWLS; Diener et al., 1985; e.g., “In most ways my life is close to ideal”). Participants indicated their agreement using a 6-point scale (1 = *Completely disagree*; 6 = *Completely agree*; *α* = 0.91).

Single Satisfaction was assessed using two items from the Satisfaction with Relationship Status Scale (Lehmann et al., 2015); e.g., “To what extent does being single meet your expectations?” Participants answered using a 4-point scale (1 = *Not at all*; 4 = *Completely*). The two questions were positively correlated (*r* = 0.72, *p* < 0.001) and averaged to form a composite score.

##### 2.2.2.2 Ill-being

Anxiety was assessed using the seven-item General Anxiety Disorder measure (GAD; Spitzer et al., 2006; e.g., “feeling nervous, anxious, or on edge”). Participants answered using a 4-point scale to indicate how often they experienced each symptom (1 = *Not at all*; 4 = *Nearly every day*; *α* = 0.94).

Depression was assessed using six-items from the mental health screening scales for the U. S. National Health Interview study (Kessler and Mroczek, 1992; e.g., “During the last 30 days, how often did you feel so sad that nothing could cheer you up?”). Participants answered using a 5-point scale (1 = *None of the time*; 5 = *All of the time*) to indicate how often each item was true for them (*α* = 0.92).

Loneliness was assessed using a short-form measure of loneliness (Hays and DiMatteo, 1987; e.g., “I feel isolated from others”). Participants rated eight items using a 4-point scale (1 = *I often feel this way*; 4 = *I never feel this way*). Items were reverse scored so that higher scores indicated greater loneliness (*α* = 0.84).

Chronic stress was assessed using a global measure of perceived stress (Cohen et al., 1983; e.g., “How often have you felt that you were unable to control the most important things in your life?”). Participants rated their response on a 5-point scale (1 = *Never*; 5 = *Very Often*; *α* = 0.84).

##### 2.2.2.3 Personality

Extraversion was assessed using eight items from John and Srivastava’s (1999) version of the Big Five Inventory (e.g., “I see myself as someone who is full of energy”). Participants rated their agreement using a 5-point scale (1 = *Strongly disagree*; 5 = *Strongly agree; α* = 0.85).

Neuroticism was assessed using eight items from Goldberg’s (2019) International Personality Item Pool (IPIP; e.g., “I get stressed out easily”). Participants rated how well each item described their personality using a 4-point scale (1 = *Not at all like me*; 4 = *Very much like me*; α = 0.91).

Self-esteem was assessed using four items from the Rosenberg Self-Esteem Scale (RSES; Rosenberg, 1965; e.g., “On the whole, I am satisfied with myself”). Participants rated their agreement with each item using a 6-point scale (1 = *Strongly disagree*; 6 = *Strongly agree*; α = 0.77).

Preference for solitude was assessed using the Preference for Solitude Scale (PSS; Burger, 1995), which consists of twelve items. Each item asked participants to choose between two statements, selecting the one that best described them (e.g., “I enjoy being around people / I enjoy being by myself”). Higher scores indicated greater preference for solitude (α = 0.78).

##### 2.2.2.4 Socialization

Friendship satisfaction was assessed using twelve items from the Friendship Network Satisfaction Scale (FNSS; Kaufman et al., 2021). Each item (e.g., “I spend a lot of time socializing with my friends”) was rated on a 5-point scale (1 = *Not at all agree*; 5 = *Completely agree*; α = 0.95).

Family satisfaction was assessed using the ten-item Family Satisfaction Scale (FSS; Olson, 1982). Participants rated their level of satisfaction with different aspects of their family (e.g., “the degree of closeness between family members”) using a 6-point scale (1 = *Not at all satisfied*, 6 = *Completely satisfied*; α = 0.96).

Emotional support was assessed using a six-item measure assessing different aspects of support (e.g., “Are there people who you can really count on to listen to you when you need to talk?”). Participants selected either 1 = *Yes* or 0 = *No* for each question. Higher scores indicated greater emotional support (*α* = 0.88).

##### 2.2.2.5 Demographics

We also assessed demographic characteristics, including gender (1 = *Male*; 2 = *Femal*e), age (18–65), race/ethnicity, income, education, and marital history. Race/ethnicity was assessed with a multi-select item (e.g., Asian, Black/African American, Hispanic/Latinx, White). Income was assessed on a 6-point scale (1 = < $30,000; 6 = ≥ $150,000) and recoded into three groups: (1) < $50,000, (2) $50,000–$100,000, (3) > $100,000. Education was also assessed on a 6-point scale (1 = *Less than high school*; 6 = *Prefer not to answer*) and recoded into three groups: (1) High school, (2) College, (3) Postgraduate. Marital history of our single participants was reported as *widowed*, *divorced*, *separated*, or *never married*. Additionally, we also included binary indicators for current life circumstances—living alone, raising a child, and attending school (1 = *Yes*; 0 = *No*).

##### 2.2.2.6 Relationship history

Relationship history was measured with a multi-select item listing seven options, each coded as a binary variable (1 = *Yes*; 0 = *No*). Options included: (1) monogamous relationships, (2) polyamorous relationships, (3) uncommitted casual dating, (4) uncommitted sexual relationships (e.g., friends with benefits), (5) one-time sexual encounters (e.g., one-night stands), and (6) no prior romantic involvement. Those selecting “no prior romantic involvement” could not choose other options.

### 2.3 Analytic strategy

To disentangle heterogeneity and identify subgroups of singles, we performed latent profile analysis (LPA) using Mplus (v. 8.10; Muthén and Muthén, 1998-2018). Model fit was evaluated using three separate categories of fit statistics—information criteria, likelihood ratio tests, and entropy.

#### 2.3.1 Information criteria

Information criteria metrics included the Akaike Information Criterion (AIC), Bayesian Information Criterion (BIC), and sample-size adjusted Bayesian Information Criterion (aBIC). Profiles with lower AIC, BIC, and aBIC statistics represent a better fit (Nylund-Gibson and Choi, 2018). Typically researchers look for a point in the progression of profile solutions where information criteria hit a nadir and then rebound; if information criteria rise when comparing a profile solution with a given number of profiles (*k*) to its predecessor (*k-1*), that suggests that adding the new profile decreases model fit, and the *k-1* profile solution is preferred.

#### 2.3.2 Likelihood tests

Tests of likelihood such as the Lo–Mendell–Rubin test (LMRT) and the Vuong-Lo–Mendell–Rubin Likelihood Ratio Test (VLMRT) statistically assess whether a solution with a given number of profiles (*k*) is an improvement over its predecessor (*k-1*). For example, if VLMRT and LMRT tests show nonsignificant results when comparing a four- vs. three-profile solution, this supports retaining the three-profile solution (Nylund et al., 2007).

#### 2.3.3 Entropy

Entropy assesses the degree of profile separation—higher entropy values indicate a greater level of profile distinctness and less overlap. While entropy metrics are not used as frequently for direct comparisons of models, they offer valuable information for assessing model quality (Nylund-Gibson and Choi, 2018).

#### 2.3.4 Enumeration of profiles

When comparing profile solutions, it is common for fit metrics to conflict with each other, offering support for competing models (Nylund-Gibson and Choi, 2018). In situations where fit indices support multiple models, solution selection should consider a combination of fit indices, classification diagnostics, and theoretical considerations such as interpretability and utility of the model (see Nylund-Gibson and Choi, 2018; Masyn, 2013).

Once an optimal solution is selected, the resulting profiles are compared to determine if there are differences in levels of outcomes and covariates; this is accomplished using the Block, Croon, and Hagenaars (BCH) approach for continuous variables, and the DCAT function for categorical variables.

To facilitate comparisons between profiles for outcomes and covariates we divided the profiles into three separate “tiers” according to their scores. The highest tier consisted of the highest-scoring profile and any other profiles that were not significantly different from it. Similarly, the lowest tier consisted of the lowest-scoring profile and any other profiles that were not significantly different from it. The profiles that did not fit into either the highest or lowest tier were assigned to the middle tier. These are discussed further in the results section.

## 3 Results

### 3.1 Latent profile analysis

Fit indices for the LPAs can be seen in Table 3. A series of ten models were estimated and the fit indices for each model compared. Information criteria (AIC, BIC and aBIC) all decreased continuously up until the nine-profile solution and then increased for the ten-profile model, indicating that the nine-profile solution was the best fit for the data. Likelihood tests (VLMRT and LMRT) were significant for all models except two, indicating (1) the three-profile solution was not a substantive improvement over the two-profile solution (VLMRT: *p* = 0.091, LMRT: *p* = 0.089), and (2) the ten-profile solution was not a substantive improvement over the nine-profile solution (VLMRT: *p* = 1.00, LMRT: *p* = 1.00).

**Table 3:** Model fit indices for latent profile analyses.

Model/Solution	-2LL	AIC	BIC	aBIC	LMRT	VLMRT	Entropy
1-Profile	72322.38	72346.38	72424.19	72386.05	—	—	—
2-Profile	65232.19	65276.19	65418.83	65348.92	<0.0001	<0.0001	1.000
3-Profile	62978.55	63042.55	63250.03	63148.35	0.0893	0.0912	0.960
4-Profile	62633.20	62717.20	62989.52	62856.06	<0.0001	<0.0001	0.824
5-Profile	62298.01	62402.01	62739.16	62573.92	<0.0001	<0.0001	0.868
6-Profile	61674.09	61798.09	62200.08	62003.06	<0.0001	<0.0001	0.885
7-Profile	61467.56	61611.56	62078.38	61849.59	0.0053	0.0057	0.859
8-Profile	61278.79	61442.79	61974.45	61713.88	0.0002	0.0002	0.873
**9-Profile**	**58955.28**	**59139.28**	**59735.77**	**59443.43**	**<0.0001**	**<0.0001**	**0.888**
10-Profile	60614.28	60818.28	61479.62	61155.50	1.00	1.00	0.921

-2LL, -2 Loglikelihood value; AIC, Akaike Information Criterion; BIC, Bayesian Information Criterion; aBIC, Adjusted Bayesian Information Criterion; LRMT, Lo–Mendell–Rubin Test; VLMRT, Vuong-Lo–Mendell–Rubin Likelihood Ratio Test. Bold values represent the best fitting model/solution.

Indices therefore offered competing support for a two-profile solution and a nine-profile solution. The two profiles were further inspected and compared. Inspection of the two-profile solution revealed a clear problem; groups of casual-daters and partner-seekers were collapsed into a single profile, even though there was greater variation in the dataset. Although individuals were allowed to select both casual dating *and* partner seeking together, only a relatively small group did so (*n* = 538; 12.2%). Larger numbers preferred *only* casual dating (*n* = 766; 15.8%) or *only* partner seeking (*n* = 1712; 35.4%). The remaining participants indicated they were not looking for either casual dates or a partner (*n* = 1819; 37.6%).

Therefore, the two-profile solution was rejected for several reasons: First, it collapsed casual-daters and partner-seekers into a single profile despite their clear divergence in the dataset. Second, accepting the two-profile solution would have been the equivalent of conducting a simple linear comparison of non-daters to everyone else, limiting the insight to be gained. Finally, extant theory on the lived experience of singles suggests there are important distinctions to be made between those seeking partners, those seeking casual dates, and those not seeking either. Kislev (2023) has argued that casual-daters form an important sub-category of singles who possess a liminal identity that distinguishes them from partner-seekers. Overall, then, the two-profile solution seemed inconsistent with the growing base of evidence supporting significant levels of heterogeneity in the single population.

In contrast, the nine-profile solution was not only congruent with the growing base of evidence for heterogeneity in the single population—it was also better supported by fit indices. Both the information criteria and likelihood tests optimally converged at the nine-profile solution, while clashing at the two-profile solution. The nine-profile solution also demonstrated high class-separation, with an entropy value of 0.90. Based on these considerations, the nine-profile solution was retained.

### 3.2 Extracted profiles

**Figure 1:**
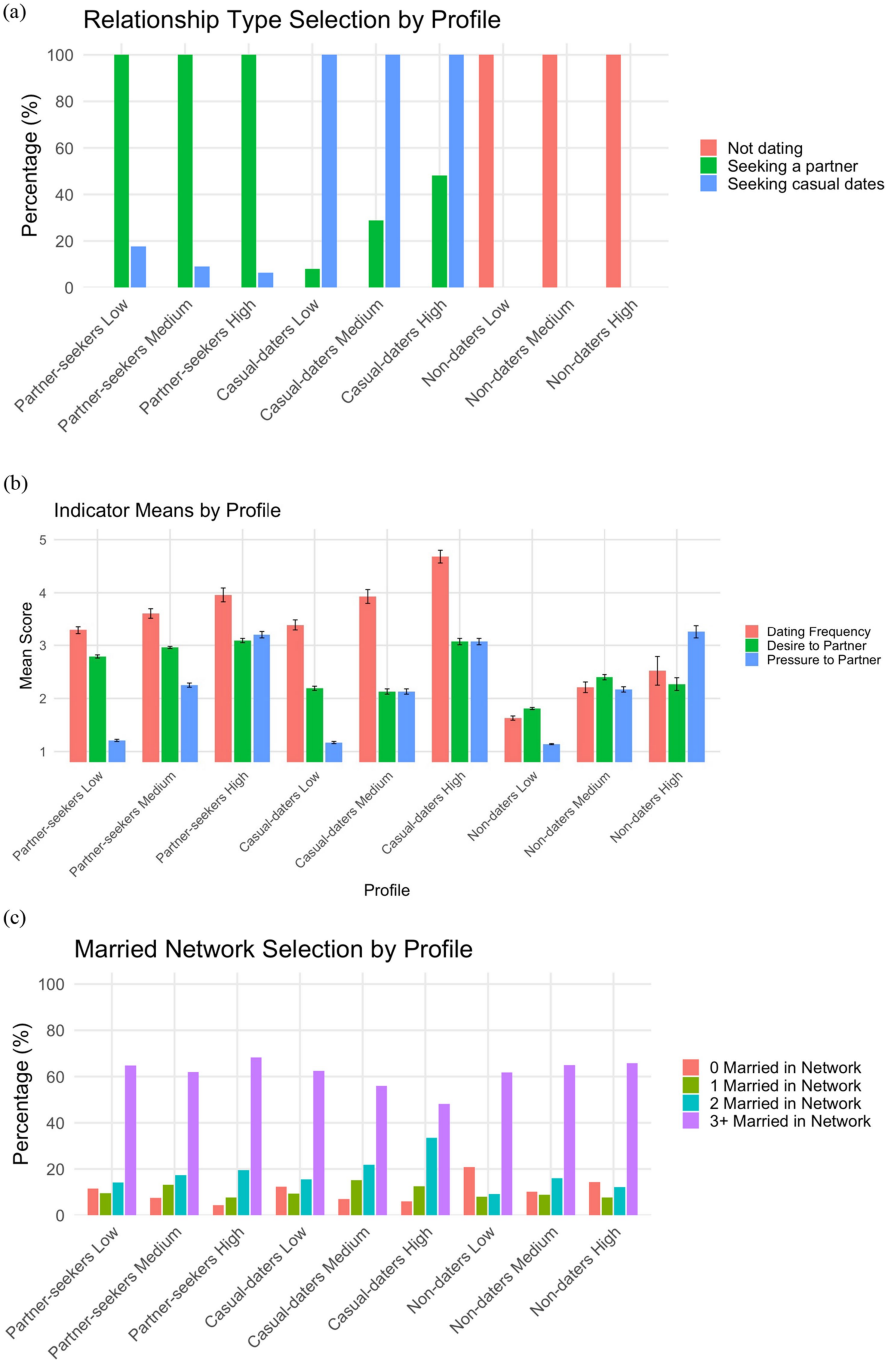
Primary Indicators by Profile. Primary indicators by profile including: **(a)** percent who selected different relationship types, such as “seeking a partner,” “seeking casual dates,” or “not dating”; **(b)** indicator means for desire to partner (4-point scale), dating frequency (7-point scale), and perceived pressure to partner (4-point scale; error bars indicate standard errors); and **(c)** percent who selected 0 to 3+ married people in their social network. Intrinsic factors include relationship type selection, desire to partner, and dating frequency. Extrinsic factors include perceived pressure to partner and married network selection.

The nine-profile solution was marked by an overarching pattern. Specifically, the nine profiles could be grouped into sets of three by romantic relationship goals (partner-seekers, casual-daters, and non-daters), then further divided again by perceived pressure to partner (low, medium, and high). We thus organized the profiles according to those indicators and described the other indicators (desire to partner, dating frequency, and married people in network) within each profile. See Table 4 and Figure 1 for indicators by profile.

**Table 4:** Primary indicators by profile.

Profile designation	Full sample	Partner-seekers		Casual-daters				Non-daters		
		Low pressure	Medium pressure	High pressure	Low pressure	Medium pressure	High pressure	Low pressure	Medium pressure	High pressure
Profile name		Relaxed seekers	Lonesome seekers	Complex seekers	Happy explorers	High social explorers	Stressed explorers	Happy non-daters	Introverted non-daters	Distressed non-daters
N (%)	4,835 (100%)	960 (19.9%)	871 (18.0%)	382 (7.9%)	402 (8.3%)	434 (9.0%)	340 (7.0%)	1,064 (22.0%)	309 (6.4%)	73 (1.5%)
Intrinsic factors (Desire)										
Percent seeking…										
Committed partner	52.4% (1.3)	100% (0)	100% (0)	100% (0)	8.1% (4.6)*	28.8% (9.9)*	48.1% (7.6)*	0% (0)*	0% (0)*	0% (0)*
Casual dates	30.0% (1.4)	17.7% (2.4)*	9.1% (5.4)*	6.3% (7.5)*	100% (0)	100% (0)	100% (0)	0% (0)*	0% (0)*	0% (0)*
No dating	29.9% (0.7)	0% (0)	0% (0)	0% (0)	0% (0)	0% (0)	0% (0)	100% (0)	100% (0)	100% (0)
Level of…										
Desire to partner (1–4)	2.54 (0.01)	2.79 (0.03)	2.96 (0.02)	3.09 (0.04)	2.19 (0.04)	2.13 (0.05)	3.07 (0.06)	1.81 (0.02)	2.40 (0.05)	2.27 (0.12)
Dating frequency (1–7)	3.12 (0.03)	3.29 (0.07)	3.61 (0.09)	3.96 (0.13)	3.39 (0.10)	3.93 (0.13)	4.68 (0.12)	1.63 (0.04)	2.21 (0.10)	2.52 (0.27)
Extrinsic factors (Pressure)										
Level of…										
Perceived pressure to partner (1–4)	1.84 (0.02)	1.21 (0.02)	2.25 (0.04)	3.20 (0.06)	1.17 (0.02)	2.13 (0.05)	3.07 (0.06)	1.14 (0.01)	2.17 (0.05)	3.26 (0.12)
Percent with…										
No Married in network	11.6% (0.5)	11.6% (1.1)	7.5% (1.1)	4.4% (1.2)	12.4% (1.7)	7.0% (1.7)	6.0% (1.6)	20.9% (1.3)	10.2% (2.0)	14.3% (4.4)
1 Married in network	10.4% (0.4)	9.5% (1.1)	13.2% (1.3)	7.7% (1.6)	9.4% (1.6)	15.2% (2.2)	12.5% (2.3)	8.1% (0.9)	8.9% (1.8)	7.7% (3.3)
2 Married in network	16.4% (0.5)	14.2% (1.3)	17.4% (1.6)	19.6% (2.6)	15.6% (2.0)	21.9% (2.5)	33.5% (3.5)	9.2% (0.9)	16.0 (2.3)	12.2% (4.1)
3+ Married in network	61.8% (0.7)	64.8% (1.8)	61.9% (1.9)	68.3% (3.4)	62.5% (2.7)	55.9% (3.0)	48.1% (4.6)	61.8% (1.5)	65.0% (3.0)	65.8% (6.0)

Cells presented as %(SE) or M(SE). *Participants were able to select both “Committed Partner” and “Casual Dates” but could not select either if they chose “Single Only.” Thus, in the Partner-seeker and Casual-dater groups, LPA assigned posterior probabilities to both the “Committed Partner” and “Casual Dates” option. All category members for Partner-seekers (100%) were coded as interested in their primary category (committed partner), and all category members (100%) for Casual-daters were coded as interested in their primary category (casual dates). The percentage for the secondary category indicates the number of participants who also selected that category. Higher percentages in a secondary category, therefore, indicate a greater group-level likelihood to cross over and express interest in the other relationship goal. Percentages may therefore sum to more than 100%. All scales are oriented such that higher numbers equal higher endorsement. Percentages and Standard errors for Relationship Type (Percent seeking…) and Relationship Network (Percent with…) variables taken from LPA analysis.

#### 3.2.1 Partner-seekers (45.8% of the full sample)

##### 3.2.1.1 Low-pressure partner-seekers (relaxed seekers)

This profile comprised 19.9% of the full sample (*n* = 960). All participants in this profile expressed a desire for a committed relationship, however, an additional 17.7% also expressed a desire to date casually. Of the partner-seeker profiles, the low-pressure profile reported both the lowest desire to partner (*M* = 2.79, *SE* = 0.03) and the lowest dating frequency (*M* = 3.29, *SE* = 0.07). This profile also had the highest proportion of participants reporting no married people in their social network (11.6%). However, many low-pressure partner-seekers also reported having 3+ married people in their network (64.8%), which was more than medium-pressure partner-seekers.

##### 3.2.1.2 Medium-pressure partner-seekers (lonesome seekers)

This profile comprised 18% of the sample (*n* = 871). As with other partner-seeker profiles, these participants expressed a desire for a committed relationship. Only 9.1% expressed an additional desire to date casually. These participants also had a moderate desire to partner (*M* = 2.96, *SE* = 0.02), moderate dating frequency (*M* = 3.61, *SE* = 0.09), the highest percentage of people reporting 1 married person in their network (13.2%), and the second highest percentage of people reporting 2 married people in their social network (17.4%).

##### 3.2.1.3 High-pressure partner-seekers (complex seekers)

This profile comprised 7.9% of the sample (*n* = 382). All participants in this profile expressed a desire for a committed relationship. The profile also had the lowest number of people (6.3%) who expressed an additional desire to date casually. Compared to the low-pressure and medium-pressure partner-seekers, participants in this profile expressed both the highest desire to partner (*M* = 3.09, *SE* = 0.04) and the highest dating frequency (*M* = 3.96, *SE* = 0.13). Additionally, their social networks skewed towards married people; this profile had the lowest percentage of participants reporting either no married (4.4%) or 1 married (7.7%) person in their network, as well as the highest number of people reporting 2 married (19.6%) and 3+ married (68.3%) people.

#### 3.2.2 Casual-daters (24.3% of sample)

##### 3.2.2.1 Low-pressure casual-daters (happy explorers)

This profile comprised 8.3% of the full sample (*n* = 402). While all participants in this profile indicated they were seeking casual dates, an additional 8.1% indicated they were also interested in finding a committed romantic partner. Compared to the medium-pressure and high-pressure casual-daters, participants in this profile expressed a low desire to partner (*M* = 2.19, *SE* = 0.04), comparable to the medium-pressure profile but substantially below the high-pressure profile. Participants in this profile also expressed the lowest dating frequency (*M* = 3.39, *SE* = 0.10) which was substantially below the other two profiles. In terms of the number of married people in their social network, singles in the low-pressure casual-dater profile not only had the highest number of people reporting no married people (12.4%), but also the highest number of people reporting 3+ married people (62.7%).

##### 3.2.2.2 Medium-pressure casual-daters (high-social explorers)

This profile comprised 9.0% of the sample (*n* = 434). As with the other casual-dater profiles, all participants in this profile indicated they were seeking casual dates. However, an additional 28.8% also indicated they were interested in finding a long-term partner. Participants in this profile had roughly the same level of desire to partner (*M* = 2.13, *SE* = 0.05) as those in the low-pressure profile; they also had a moderate level of dating frequency (*M* = 2.13, *SE* = 0.05) compared to the low-pressure and high-pressure casual-dater profiles. In terms of social network composition, they were relatively unremarkable compared to other casual-daters.

##### 3.2.2.3 High-pressure casual-daters (stressed explorers)

This profile comprised 7% of the sample (*n* = 340). As with the previous two profiles, participants in this high-pressure profile all indicated they were seeking casual dates, but nearly half of them (48.1%) also sought a long-term romantic partner. Relative to the other casual-dater profiles, participants in this profile indicated a markedly greater desire to partner (*M* = 3.07, *SE* = 0.06) and a greater dating frequency (*M* = 4.68, *SE* = 0.12). Additionally, participants in this profile were the least likely to have no married people (6%) or 3+ married people (48.1%) in their network but were also most likely to have 2 married people (33.5%) in their network.

#### 3.2.3 Non-daters (29.8% of sample)

##### 3.2.3.1 Low-pressure non-daters (happy non-daters)

This profile comprised 22% of the full sample (*n* = 1,064) and was the largest of the extracted profiles. Relative to participants in the other non-dater profiles, the participants in the low-pressure non-daters profile exhibited the lowest desire to partner (*M* = 1.81, *SE* = 0.02) and the lowest dating frequency (*M* = 1.63, *SE* = 0.04). Additionally, 20.9% reported having no married people in their network.

##### 3.2.3.2 Medium-pressure non-daters (introverted non-daters)

This profile comprised 6.3% of the sample (*n* = 309). Participants in the medium-pressure non-daters profile showed a higher desire to partner (*M* = 2.40, *SE* = 0.05) than those in the low-pressure profile, but not the high-pressure profile. In terms of dating frequency, the participants in this profile also had a moderate score (*M* = 2.21, *SE* = 0.10), falling between the low and high-pressure profiles. Finally, in terms of their social networks, this profile had the lowest number of participants reporting no married people (10.2%) compared to the other non-dater profiles, and the highest number reporting 2 married people (16.0%).

##### 3.2.3.3 High-pressure non-daters (distressed non-daters)

This profile comprised only 1.5% of the sample (*n* = 73) and was the smallest of the nine extracted profiles. Relative to the other non-dater profiles, participants in the high-pressure non-daters profile showed a higher desire to partner (*M* = 2.27, *SE* = 0.12) than those in the low-pressure profile—but not the medium-pressure profile. This profile also showed the highest levels of dating frequency (*M* = 2.52, *SE* = 0.27). In terms of the number of married people in their network, participants in the high-pressure profile were not particularly remarkable; in most cases they fell somewhere between those in the low and high-pressure profiles.

### 3.3 Outcomes and covariates

Table 5 presents profile comparisons for psychological outcomes and covariates. To facilitate comparisons, profiles have been divided into three tiers for each variable. Only the top and bottom tiers are discussed, as those represent the extreme edges of the profile distribution and allow for a discussion of which profiles are exceptional in some way.

**Table 5:** Psychological outcomes and covariates by profile.

Profile designation	Full sample	Partner-seekers			Casual-daters			Non-daters		
		Low pressure	Medium pressure	High pressure	Low pressure	Medium pressure	High pressure	Low pressure	Medium pressure	High pressure
Profile name		Relaxed seekers	Lonesome seekers	Complex seekers	Happy explorers	High social explorers	Stressed explorers	Happy non-daters	Introverted non-daters	Distressed non-daters
N (%)	4,835 (100%)	960 (19.9%)	871 (18.0%)	382 (7.9%)	402 (8.3%)	434 (9.0%)	340 (7.0%)	1,064 (22.0%)	309 (6.4%)	73 (1.5%)
Well-Being										
Life Satisfaction (1–6)	3.85 (0.02)	3.81 (0.04) ^2^	3.69 (0.04) ^2^	3.75 (0.06) ^2^	4.10 (0.06) ^1^	3.96 (0.06) ^1^	4.02 (0.07) ^1^	3.97 (0.04) ^1^	3.60 (0.07) ^2^	3.27 (0.14) ^3^
Single Satisfaction (1–4)	2.89 (0.01)	2.69 (0.03) ^2^	2.44 (0.03) ^3^	2.46 (0.04) ^3^	3.21 (0.04) ^1^	3.01 (0.04) ^2^	2.83 (0.05) ^2^	3.41 (0.02) ^1^	3.04 (0.05) ^2^	3.10 (0.12) ^2^
Ill-Being										
Anxiety (1–4)	1.88 (0.01)	1.71 (0.03) ^2^	2.07 (0.03) ^2^	2.29 (0.05) ^1^	1.55 (0.04) ^3^	1.99 (0.05) ^2^	2.40 (0.05) ^1^	1.56 (0.03) ^3^	2.02 (0.05) ^2^	2.42 (0.11) ^1^
Depression (1–5)	2.24 (0.01)	2.02 (0.04) ^2^	2.50 (0.04) ^2^	2.71 (0.06) ^1^	1.79 (0.05) ^3^	2.43 (0.06) ^2^	2.92 (0.06) ^1^	1.79 (0.03) ^3^	2.43 (0.06) ^2^	3.00 (0.14) ^1^
Loneliness (1–4)	2.43 (0.01)	2.40 (0.03) ^2^	2.63 (0.03) ^2^	2.70 (0.04) ^1^	2.15 (0.04) ^3^	2.38 (0.04) ^2^	2.69 (0.04) ^1^	2.16 (0.02) ^3^	2.62 (0.04) ^2^	2.77 (0.09) ^1^
Stress (Chronic) (1–5)	3.10 (0.01)	3.01 (0.02) ^3^	3.16 (0.02) ^2^	3.29 (0.03) ^1^	3.05 (0.03) ^2^	3.15 (0.03) ^2^	3.30 (0.04) ^1^	2.97 (0.02) ^3^	3.13 (0.03) ^2^	3.28 (0.07) ^1^
Personality										
Extraversion (1–5)	3.00 (0.01)	3.07 (0.03) ^2^	3.01 (0.03) ^2^	3.07 (0.04) ^2^	3.08 (0.04) ^2^	3.12 (0.04) ^1^	3.20 (0.04) ^1^	2.87 (0.02) ^2^	2.82 (0.05) ^3^	2.68 (0.09) ^3^
Neuroticism (1–4)	2.49 (0.01)	2.35 (0.03) ^2^	2.65 (0.02) ^2^	2.77 (0.04) ^1^	2.22 (0.04) ^3^	2.50 (0.04) ^2^	2.79 (0.04) ^1^	2.27 (0.02) ^3^	2.66 (0.04) ^2^	2.92 (0.07) ^1^
Self-Esteem (1–6)	4.21 (0.02)	4.40 (0.04) ^2^	3.91 (0.04) ^2^	3.87 (0.06) ^2^	4.69 (0.06) ^1^	4.12 (0.07) ^2^	3.72 (0.07) ^3^	4.59 (0.04) ^1^	3.95 (0.08) ^2^	3.42 (0.15) ^3^
Pref. for Solitude	1.64 (0.00)	1.63 (0.01) ^2^	1.59 (0.01) ^2^	1.58 (0.02) ^2^	1.68 (0.01) ^2^	1.58 (0.02) ^3^	1.52 (0.02) ^3^	1.74 (0.01) ^1^	1.70 (0.02) ^2^	1.72 (0.03) ^1^
Connection										
Emotional Support	3.92 (0.02)	4.07 (0.06) ^1^	3.88 (0.06) ^2^	3.84 (0.09) ^2^	4.17 (0.08) ^1^	4.01 (0.09) ^1^	3.55 (0.11) ^3^	3.96 (0.06) ^2^	3.82 (0.11) ^2^	2.89 (0.25) ^3^
Family Satisfaction (1–6)	3.87 (0.02)	3.85 (0.05) ^2^	3.79 (0.05) ^2^	3.76 (0.07) ^2^	4.12 (0.08) ^1^	3.90 (0.07) ^2^	4.04 (0.08) ^1^	3.97 (0.05) ^1^	3.64 (0.09) ^3^	3.13 (0.17) ^3^
Friend Satisfaction (1–6)	3.33 (0.02)	3.30 (0.04) ^2^	3.41 (0.04) ^2^	3.51 (0.05) ^1^	3.47 (0.06) ^1^	3.55 (0.06) ^1^	3.62 (0.06) ^1^	3.06 (0.04) ^3^	3.20 (0.07) ^3^	2.97 (0.15) ^3^

Cells presented as M(SE). Means and Standard Errors taken from BCH analysis. Superscripts 1–3 indicate tiers: 1 = the highest tier consisting of the highest-scoring profile on a given variable and any other profiles that were not significantly different from it. 3 = the lowest tier consisting of the lowest-scoring profile and any other profiles that were not significantly different from it. 2 = the middle tier consisting of profiles that did not fit into either the highest or lowest tiers.

#### 3.3.1 Primary outcomes

##### 3.3.1.1 Life satisfaction

The highest tier of life satisfaction included all three casual-dater profiles and the low-pressure non-dater profile. The highest levels of life satisfaction were reported by the low-pressure casual-daters (*M* = 4.10, *SE* = 0.06), followed by high-pressure casual-daters (*M* = 4.02, *SE* = 0.07), then low-pressure non-daters (*M* = 3.97, *SE* = 0.04) and medium-pressure casual-daters (*M* = 3.96, *SE* = 0.06). Only one profile was in the lowest tier of life satisfaction: high-pressure non-daters (*M* = 3.27, *SE* = 0.14).

##### 3.3.1.2 Satisfaction with singlehood

Satisfaction with singlehood was the only variable where it was necessary to define four tiers. Tiers one and two were both high satisfaction, tier three was moderate, and tier four was low satisfaction. The only profile on tier one (high satisfaction) included the low-pressure non-daters (*M* = 3.41, *SE* = 0.02). The second tier included low-pressure casual-daters (*M* = 3.21, *SE* = 0.04) and high-pressure non-daters (*M* = 3.10, *SE* = 0.12). The lowest tier (low satisfaction) included medium-pressure partner-seekers (*M* = 2.44, *SE* = 0.03) and high-pressure partner-seekers (*M* = 2.46, *SE* = 0.04).

#### 3.3.2 Psychological covariates

##### 3.3.2.1 Ill-being

The four measures of ill-being (anxiety, depression, loneliness, and chronic stress) were so closely related across profiles that they can be discussed together. The highest tier of ill-being included the three high-pressure profiles. Regardless of the specific variable used to index ill-being, the high-pressure profiles scored the highest and were not significantly different from each other.

The lowest tier of ill-being included the three low-pressure profiles, with some minor deviations. Low-pressure non-daters were at the lowest tier of ill-being regardless of the measure used. Low-pressure casual-daters were in the lowest tier of ill-being for all variables except chronic stress, which was occupied by low-pressure partner-seekers.

##### 3.3.2.2 Personality

The highest tier of neuroticism consisted of the three high-pressure profiles: high-pressure partner-seekers (*M* = 2.77, *SE* = 0.04), high-pressure casual-daters (*M* = 2.79, *SE* = 0.04) and high-pressure non-daters (*M* = 2.92, *SE* = 0.07). The lowest tier of neuroticism contained low-pressure profiles, including low-pressure casual-daters (*M* = 2.22, *SE* = 0.04) and low-pressure non-daters (*M* = 2.27, *SE* = 0.02).

Self-esteem was similarly related to pressure, but the direction of the effect was reversed. The highest tier of self-esteem contained low-pressure profiles, including low-pressure casual-daters (*M* = 4.69, *SE* = 0.06) and non-daters (*M* = 4.59, *SE* = 0.04), while the lowest tier of self-esteem contained high-pressure profiles, including high-pressure casual-daters (*M* = 3.72, *SE* = 0.07) and high-pressure non-daters (*M* = 3.42, *SE* = 0.15).

The highest tier of extraversion consisted of the high-pressure (*M* = 3.20, *SE* = 0.04) and medium-pressure (*M* = 3.12, *SE* = 0.04) casual-daters. The lowest tier consisted of the high-pressure (*M* = 2.68, *SE* = 0.09) and medium-pressure (*M* = 2.82, *SE* = 0.05) non-daters.

The highest tier of preference for solitude consisted of the low-pressure (*M* = 1.74, *SE* = 0.01) and the high-pressure (*M* = 1.72, *SE* = 0.03) non-daters, while the lowest tier of preference for solitude consisted of the high-pressure casual-daters (*M* = 1.52, *SE* = 0.02).

##### 3.3.2.3 Socialization

The highest tier of friendship satisfaction consisted of high-pressure partner-seekers (*M* = 3.51, *SE* = 0.05), as well as low-pressure (*M* = 3.47, *SE* = 0.06), medium-pressure (*M* = 3.55, *SE* = 0.06) and high-pressure (*M* = 3.62, *SE* = 0.06) casual-daters. The lowest tier of friendship satisfaction contained the three non-dater profiles—low-pressure (*M* = 3.06, *SE* = 0.04), medium-pressure (*M* = 3.20. *SE* = 0.07) and high-pressure (*M* = 2.97, *SE* = 0.15) All non-daters reported experiencing the lowest levels of friendship satisfaction, which were not significantly different from each other.

The highest tier of family satisfaction consisted of low-pressure casual-daters (*M* = 4.12, *SE* = 0.08), high-pressure casual-daters (*M* = 4.04, *SE* = 0.08), and low-pressure non-daters (*M* = 3.97, *SE* = 0.05). The lowest tier of family satisfaction consisted of high-pressure non-daters (*M* = 3.13, *SE* = 0.17).

The highest tier of emotional support consisted of low-pressure partner-seekers (*M* = 4.07, *SE* = 0.06), as well as low-pressure (*M* = 4.17, *SE* = 0.08) and medium-pressure (*M* = 4.01, *SE* = 0.09) casual-daters. The lowest tier of emotional support consisted of high-pressure non-daters (*M* = 2.89, *SE* = 0.25).

#### 3.3.3 Demographic covariates

##### 3.3.3.1 Standard demographics

In additional to the full sample demographics, Table 1 contains demographic information by profile.

##### 3.3.3.2 Gender

A clear pattern of gender differences emerged across profiles. Female participants were over-represented in non-dater profiles; the low-pressure (67.6%), medium-pressure (71.2%) and high-pressure (69.8%) non-dater profiles all reported the highest percentages of women. High-pressure partner-seekers also had a high percentage of women (64.2%). In all these profiles, the percentages of women were higher than the full sample norm (57.5%). Conversely, male participants were over-represented in casual-dater profiles; the low-pressure (57.0%), medium-pressure (53.2%), and high-pressure (55.2%) profiles had the highest proportion of males, along with low-pressure partner-seekers (50.6%). Again, in all cases, they were higher percentages than the full sample norm (42.5%). The general pattern for gender, then, is that non-daters tend to skew female, casual-daters tend to skew male, and partner-seekers skew male when low pressure and female when high-pressure.

##### 3.3.3.3 Age

Clear patterns of age differences also emerged across profiles. The highest proportion of 45–65-year-olds appeared in the low-pressure non-daters (77.6%), low-pressure casual-daters (65.4%) and low-pressure partner-seekers (55.6%). Conversely, the lowest proportion of 46–65-year-olds appeared in the high-pressure casual-daters (10.0%), followed by high-pressure partner-seekers (18.1%), then high-pressure non-daters (22.1%). Inverse patterns appeared among 18–24-year-olds, although they were less clear. They were most evident in the low-pressure profiles; the lowest proportion of 18–24-year-olds were found in the low-pressure non-daters (6.6%), low-pressure casual-daters (14.8%), and low-pressure partner-seekers (14.9%). Similarly, the lowest proportion of 25–45-year-olds were found in the low-pressure non-daters (15.9%) followed by low-pressure casual-daters (19.7%) and low-pressure partner-seekers (29.5%). The general pattern for age was an inverse relationship between age and pressure to partner, such that individuals past prime marriage and reproductive years were likely to report less pressure to partner.

##### 3.3.3.4 Race/ethnicity

Unlike the age and gender demographics, patterns for race/ethnicity were less clear. Only a few patterns emerged. First, Hispanic/Latinx individuals tended to be over-represented in the medium- and high-pressure categories and under-represented in the low-pressure categories. Second, there appeared to be a consistent inverse relationship between pressure and race/ethnicity, such that White/Caucasian people were more likely to belong to low-pressure profiles. The highest proportion of White participants was in the low-pressure non-daters (82.1%) followed by low-pressure partner-seekers (76.2%) and then low-pressure casual-daters (74.3%). There did not appear to be an easily identifiable consistent pattern for either Asian or Black/African American participants.

##### 3.3.3.5 Socioeconomic demographics

In terms of socioeconomic demographics, there were no clear patterns for education. However, we did see patterns for income, prior marital status, and household characteristics. The socioeconomic demographics for all profiles can be seen in Table 2 alongside demographics for the full sample.

###### 3.3.3.5.1 Income

The highest proportion of participants reporting annual earnings below $50,000 was in the low-pressure non-dater (61.6%) and high-pressure non-dater (59.8%) profiles. The lowest proportion of participants reporting earnings below $50,000 was in the high-pressure casual-dater profile (27.6%). The lowest proportion of participants reporting earnings over $100,000 were in the low-pressure (8.2%) and medium-pressure (10.2%) non-dater profiles, as well as the low-pressure (10.8%) casual-daters. The general pattern for income seems to be driven by two factors. First, for both partner-seekers and casual-daters, income is positively related to perceived pressure to partner. Second, non-daters tend to have lower income overall, as well as no clear pattern between their income and perceived pressure to partner.

###### 3.3.3.5.2 Prior marital status

Low-pressure non-daters (12.8%) and low-pressure casual-daters (8.7%) had the highest percentages of widowed people by a wide margin, relative to other profiles. Similarly, the percentage of divorced people were highest in the low-pressure partner-seeker (27.9%), low-pressure non-daters (26.8%), and low-pressure casual-dater (24.9%) profiles. Conversely, the lowest proportion of never-married individuals was found in the low-pressure non-daters (56.8%), low-pressure casual-daters (64.2%), and low-pressure partner-seekers (64.2%). Across profiles, the general pattern suggested a prior marriage was related to reduced pressure to partner. The exception to this was for those who reported being “separated”—there was no clear, consistent pattern of results across profiles for those individuals.

###### 3.3.3.5.3 Household characteristics

There did not appear to be any consistent pattern among profiles for participants who reported living alone. There was, however, a clear pattern for those attending school. The lowest levels of school attendance were reported by low-pressure non-daters (4.2%), low-pressure casual-daters (9.4%), and low-pressure partner-seekers (10.4%). The pattern, then, appeared to be that attending school was associated with medium-to-high levels of pressure to partner, perhaps due to age differences among the profiles.

There were hints of links between perceived pressure to partner and having a child in the household, but they were unclear and inconsistent. However, one notable finding did emerge. High-pressure casual-daters were the most likely to report having a child (25%), while medium-pressure casual-daters were second most likely (18.4%).

#### 3.3.4 Relationship history

See Table 6 for relationship history by profile. Some clear patterns emerged. First, in terms of those reporting a prior monogamous relationship, the highest percentages were among low-pressure partner-seekers (86.0%), high-pressure partner-seekers (82.8%), low-pressure casual-daters (81.6%), and high-pressure casual-daters (83.7%). The high-pressure non-daters had the lowest percentage of individuals reporting a prior monogamous relationship (57.9%).

**Table 6:** Relationship history by profile.

Profile designation	Full sample	Partner-seekers			Casual-daters			Non-daters		
		Low pressure	Medium pressure	High pressure	Low pressure	Medium pressure	High pressure	Low pressure	Medium pressure	High pressure
Profile name		Relaxed seekers	Lonesome seekers	Complex seekers	Happy explorers	High social explorers	Stressed explorers	Happy non-daters	Introverted non-daters	Distressed non-daters
N(%)	4,835 (100%)	960 (19.9%)	871 (18.0%)	382 (7.9%)	402 (8.3%)	434 (9.0%)	340 (7.0%)	1,064 (22.0%)	309 (6.4%)	73 (1.5%)
Romantic history contains.										
One Partner		86.0% (1.3) ^1^	77.9% (1.6) ^2^	82.8% (2.1) ^1^	81.6% (2.1) ^1^	76.2% (2.6) ^2^	83.7% (2.5) ^1^	75.4% (1.4) ^2^	70.9% (2.9) ^3^	57.9% (6.2) ^3^
Multiple Partners*		7.9% (1.0) ^2^	9.6% (1.2) ^2^	7.4% (1.5) ^3^	10.2% (1.6) ^2^	14.5% (2.2) ^1^	26.2% (2.8) ^1^	5.4% (0.7) ^3^	4.4% (1.3) ^3^	7.0% (3.2) ^3^
Casual dating		44.5% (1.8) ^2^	35.8% (1.9) ^2^	39.2% (2.7) ^2^	50.7% (2.7) ^2^	59.6% (3.1) ^2^	52.7% (3.3) ^1^	28.1% (1.4) ^3^	30.6% (3.0) ^3^	19.7% (5.1) ^3^
Uncommitted (FWB)		35.6% (1.8) ^2^	30.6% (1.8) ^2^	29.1% (2.5) ^2^	42.5% (2.7) ^2^	47.3% (3.1) ^1^	51.3% (3.3) ^1^	21.6% (1.3) ^3^	28.8% (2.9) ^2^	15.4% (4.7) ^3^
One-Time Encounters		38.6% (1.8) ^2^	30.4% (1.8) ^2^	21.3% (2.3) ^3^	41.2% (2.6) ^2^	43.5% (3.1) ^1^	50.9% (3.3) ^1^	25.0% (1.4) ^3^	26.2% (2.8) ^3^	17.1% (4.9) ^3^
No romantic involvement		5.3% (0.8) ^2^	7.3% (1.0) ^2^	5.4% (1.3) ^2^	3.7% (1.0) ^3^	4.5% (1.3) ^3^	1.7% (1.0) ^3^	17.1% (1.2) ^2^	17.5% (2.5) ^1^	31.2% (5.8) ^1^

Cells presented as %(SE). Percentages and Standard Errors taken from DCAT analysis. Superscripts 1–3 indicate tiers: 1 = the highest tier consisting of the highest-scoring profile on a given variable and any other profiles that were not significantly different from it. 3 = the lowest tier consisting of the lowest-scoring profile and any other profiles that were not significantly different from it. 2 = the middle tier consisting of profiles that did not fit into either the highest or lowest tiers.

The highest proportion of participants reporting no prior romantic involvement was in the high-pressure non-dater category (31.2%), followed by the medium-pressure (17.5%) and low-pressure (17.1%) Non-daters. The lowest proportion of participants reporting no prior romantic involvement was in the high-pressure casual-dater profile (1.7%), followed by the low-pressure casual-daters (3.7%) and the medium-pressure casual-daters (4.5%).

The other four relationship types—polyamory relationships, casual dating, friends with benefits, and one-time sexual encounters—were remarkably consistent across profiles. Generally, the pattern was that medium-pressure and high-pressure casual-daters were the most likely to report having these types of relationships previously; while low-pressure, medium-pressure, and high-pressure non-daters were the least likely.

At the broadest level, the pattern appeared to be that non-daters were the least likely to have tried any relationship and the causal daters were the most likely to have tried any relationship—occasionally by very wide margins. There were occasional deviations from this pattern (see Table 6), but the general pattern was very consistent with the partner preferences of each profile. Casual-dater profiles indicated more experimental and casual relationships, while non-dater profiles indicated disinterest in romance.

#### 3.3.5 Naming the profiles

Based on the patterns of variables across profiles we assigned names to each profile that reflect the noteworthy features of their psychological and demographic characteristics. The results can be seen in Table 7 (for partner-seeker profiles), Table 8 (for casual-dater profiles), and Table 9 (for non-dater profiles). To simplify the discussion, we refer to the profiles throughout the discussion section by the names assigned to them in Tables 7–9. Note that we have designated two profiles as possibly “single at heart” because they reflect key characteristics of DePaulo’s (2023c) description of the construct. Below, we further discuss this construct and its potential alignment with our findings.

**Table 7:** Summary of characteristics for partner-seeker profiles.

	Psychological					Demographic		Romantic history	Holistic	
Pressure	Well-Being		Ill-Being	Personality	Socialization	Gender	Age		Description	Name
Low (19.9%)	**+**				Emo Support	Male	Older	One-Partner	Skews older and male; low stress, feels emotionally supported by others, more likely to have had a one-partner relationship.	Relaxed Seekers
	**−**		Chronic stress			Female	Younger			
Medium (18.0%)	**+**		Loneliness						Almost completely unremarkable, except they are lonely and dissatisfied with singlehood	Lonesome Seekers
	**−**	Satisfaction w. Singlehood								
High (7.9%)	**+**		Anxiety Depression Loneliness Chronic Stress	Neuroticism	Friend Sat	Female	Younger	One-Partner	Skews younger and female; low single satisfaction, high ill-being, but happy with friendships. More likely to have had one-partner relationship, less likely multi-partner or promiscuous	Complex Seekers
	**−**	Satisfaction w. Singlehood				Male	Older	Polyamory One Night Stand		

**Table 8:** Summary of characteristics for casual-dater profiles.

	Psychological					Demographic		Romantic history	Holistic	
Pressure	Well-Being		Ill-Being	Personality	Socialization	Gender	Age		Description	Name
Low (8.3%)	**+**	Life Sat. Single Sat.		Self Esteem	Emo Support Family Sat Friend Sat	Male	Older	One-Partner	Skews older and male; high well-being and low-ill-being, self-assured, supported by others. More likely to have had a one-partner relationship, less likely to have had no relationship at all.	Happy Explorers (*Possibly Single at Heart)
	**−**		Anxiety Depression Loneliness	Neuroticism		Female	Younger	No History		
Medium (9.0%)	**+**	Life Sat.		Extraversion	Emo Support Friend Sat.	Male		Polyamory Casual dating FWB One Night Stand	Skews male; happy, outgoing, well supported by friends. More likely to have tried many relationships, less likely to have no relationship history.	High-Social Explorers
	**−**							No History		
High (7.0%)	**+**	Life Sat	Anxiety Depression Loneliness Chronic Stress	Extraversion Neuroticism	Family Sat. Friend Sat.	Male	Younger	One-Partner Polyamory Casual dating FWB One Night Stand	Skews younger and male; high life satisfaction but high ill-being and neuroticism, and low self-esteem. Very social, supported by friends.	Stressed Explorers
	−			Self Esteem Pref. Solitude				No History		

**Table 9:** Summary of characteristics for non-dater profiles.

	Psychological					Demographic		Romantic history	Holistic	
Pressure	Well-Being		Ill-Being	Personality	Socialization	Gender	Age		Description	Name
Low (22.0%)	**+**	Life Sat, Single Sat.		Self Esteem Pref. Solitude	Family Sat.	Female	Older		Skews older and female. High levels of happiness and well-being. High family satisfaction, but low friend satisfaction.	Happy Non-daters (*Possibly Single at Heart)
	**−**		Anxiety Depression Loneliness Chronic Stress	Neuroticism	Friend Sat.			Polyamory Casual dating FWB One Night Stand		
Medium (6.3%)	**+**		Loneliness			Female		No History	Skews female. Tends towards loneliness, low extraversion, low friend satisfaction (but average family satisfaction). More likely to have no relationship history, less likely to have tried any relationship category.	Introverted Non-daters
	**−**			Extraversion	Friend Sat.			One Partner Polyamory Casual Date FWB One Night Stand		
High (1.5%)	**+**	Single Sat.	Anxiety Depression Loneliness Chronic Stress	Neuroticism Pref. Solitude		Female	Younger	No History	Skews female and younger. Exceptionally unhappy overall, with low social support, low extroversion, but happy with singlehood. More likely to have no relationship history, less likely to have tried any other relationship category. Low friend and family satisfaction.	Distressed Non-daters
	**−**	Life Sat.		Self Esteem Extraversion				One Partner Polyamory Casual Date FWB One Night Stand		

## 4 Discussion

Overall, we addressed three research questions. First, can singles be effectively categorized into distinct “types”? Second, do some types of singles fare significantly better or worse than others? Finally, do different types of singles map onto recognizable lay categories or established research? We discuss our findings for these questions below.

### 4.1 Can singles be categorized into types?

Our results suggest singles can indeed be divided into types. This typology is primarily shaped by the interaction between two variables–type of relationship desired (i.e., romantic goals) and perceived pressure to partner–which form a “master framework” dividing our LPA profiles (or groups) into three romantic goal categories (partner-seekers, casual-daters, and non-daters) and three levels of pressure (high, medium, and low). The remaining indicators varied across the nine profiles but often lacked the clarity and consistency of the master framework. Together, romantic goals and perceived pressure form a conceptual grid that helps situate our other findings. While useful, the framework does not explain everything; some variables do not easily map onto it, and we advise others to consider this when applying these variables elsewhere.

Some research supports romantic goals as a key factor in singles’ experiences. Stein’s (1978) voluntary vs. involuntary distinction is closely tied to singles’ well-being (e.g., Apostolou et al., 2019) and romantic goals. For example, someone who sees their singlehood as involuntary may be more likely to seek a partner, while someone who sees it as voluntary may prefer to stay single. The romantic goals measured in this study may capture a more nuanced version of a primary dimension that singles use to define themselves, as well as how it might relate to their well-being.

There is also precedent for including stigma as a variable influencing singles’ experiences. Stigma is a potent driver of physical and psychological health (e.g., Dickerson et al., 2004), and when singles experience stigma, it likely influences how they view themselves.

Finally, our “master framework” aligns with current theoretical discussions in singlehood research. Our finding that an intrinsic motivation (romantic goals) and extrinsic motivation (perceived pressure to partner) combine to form a typology framework parallels Kislev’s (2023) work on studying singlehood as an identity. While our taxonomy does not assess identity directly, it informs aspects of Kislev’s (2023) theory. His theory suggests that those who date casually are likely to experience singlehood as a “peripheral identity” while those who avoid dating are likely to experience it as either a “core identity” (if their choice is accepted) or a “counter-normative identity” (if their choice is stigmatized). Our results partly support Kislev’s work but also deviate from it in some important ways.

First, Kislev’s “core identity” has a clear analogue in our taxonomy in the form of the Happy Non-daters (the low-pressure non-daters). Kislev’s “counter-normative identity” has a clear analogue in our taxonomy in the form of Distressed Non-daters (the high-pressure non-daters). These profiles comprise 22.0 and 1.5% of our sample, respectively. Both profiles are not dating, but the presence or absence of stigma (in the form of perceived pressure to partner) appears to influence their well-being. The accepted Happy Non-dater profile is one of the happiest in our sample, while the stigmatized Distressed Non-dater profile is the least happy.

In contrast, Kislev’s “peripheral identity” category seems to have no clear parallel in our analysis. Rather, our analysis suggests that Kislev’s “peripheral identity” category could be expanded by reconsidering the relationship between stigma, casual dating, and peripheral identity status. Our analysis suggests that some casual-dater profiles may have a relatively stable single identity marked by low internal psychological conflict, low desire to partner, and frequent casual dating to fulfill relational and sexual needs, corresponding to the Happy Explorers. They are happy with singlehood, happy overall, and experience little perceived pressure to partner. As external pressure increases, however, so does the desire to find a long-term partner. This dynamic may reflect the liminal “peripheral identity” suggested by Kislev, where a person identifies as single but is actively considering the possibility of leaving singlehood. The Stressed Explorers illustrate this ambivalent identity; although 100% expressed a desire to date casually, 48.1% also indicated a desire to find a partner.

### 4.2 Which singles fare better?

Our second question asked whether some profiles fared better or worse, and explored whether any profiles defied a simple good vs. bad distinction. On the first point, our analysis returned a relatively clear answer: Happy Non-daters (low-pressure non-daters) and Happy Explorers (low-pressure casual-daters) consistently reported the highest well-being and lowest ill-being. We refer to these profiles as possibly “single at heart” because they reflect key characteristics of DePaulo’s (2023c) description of the construct. Together, they comprise 30.3% of the full sample, suggesting a substantial portion of singles are thriving. The larger of the two profiles (the Happy Non-daters), represents 22.0% of the full sample. Importantly, both profiles tend to fare better in terms of well-being and ill-being than all the partner-seekers.

One profile stood out as faring notably worse than the others: the Distressed Non-daters (high-pressure non-daters). This group reports poor well-being and ill-being with one exception—they have moderate single satisfaction. Two possible explanations may account for this pattern. The first explanation involves external factors—Distressed Non-daters may feel content being single but face external pressure to partner, and the ongoing struggle may make them unhappy. The second explanation involves internal factors—Distressed Non-daters may exhibit an avoidant attachment style, which, despite their satisfaction with singlehood, may weaken their social connections and reduce happiness. Our data support both possibilities: Distressed Non-daters report high perceived pressure to partner (external) and low friendship and family satisfaction (internal). Future research could explore which factors predominate, or whether they interact. Although this profile makes up just 1.5% of the full sample (warranting cautious interpretation), it may reflect millions of individuals when applied at scale to the broader U.S. population.

Finally, we also identified a profile that defies the good vs. bad distinction: the Stressed Explorers (high-pressure casual-daters). This profile reports very high life satisfaction alongside elevated anxiety, depression, loneliness, and chronic stress. One possible explanation is they lead a high-risk, high-reward lifestyle, marked by excitement and exploration, but also strong perceived pressure to partner. This pattern is rare in the literature, as higher well-being tends to co-occur with lower ill-being, but these findings provide emerging evidence that the two variables can diverge (Horton et al., 2024).

#### 4.2.1 The happiest singles

Our two happiest profiles—the Happy Explorers and Happy Non-daters—seem to mirror DePaulo’s (2023c) “single at heart” individuals: those who find singlehood authentic, meaningful, and fulfilling. Her quiz, based on responses from over 8,000 people across 100 countries, assesses this identity via autonomy, decision-making, social preferences, and romantic outlook. Both profiles scored high on traits aligned with this identity, including preference for solitude and family satisfaction.

Preference for solitude stands out. While typically linked to poorer mental health (Sakurai et al., 2024; Waskowic and Cramer, 1999), both Happy Explorers and Happy Non-daters report high well-being despite high solitude scores—the highest among their respective groups. Both profiles also show low anxiety, depression, and loneliness. However, solitude alone is not sufficient: Distressed Non-daters also score high on solitude but low on well-being, highlighting that autonomy—choosing solitude—may be key.

These findings also align with Pepping et al. (2024), who argue that “single at heart” should involve low desire to partner combined with high social and psychological well-being. By that standard, Happy Explorers are a strong candidate, showing low desire to partner, high well-being, and strong social ties. Though they date casually, DePaulo (2023a) notes that being “single at heart” allows for occasional dating as long as one is not organizing their life around just one romantic partner.

Looking beyond theory, what defines the happiest singles? They fall into two types: those avoiding romantic attachment (more often women) and those casually dating (more often men). Both groups are typically older, more likely previously married, low in desire and pressure to partner, and high in well-being. Personality-wise, they are moderately extraverted, low in neuroticism, and high in self-esteem. Their main differences lie in social domains: Happy Explorers are more socially fulfilled (especially in friendships), while Happy Non-daters are mainly satisfied with family. Relationship history also differs—Happy Explorers are more likely to have tried multiple relationship types (e.g., polyamory, friends with benefits), while Happy Non-daters are among the least likely to have done so.

### 4.3 Do some singles align with lay categories or established research?

Our final question asked whether our singles profiles map onto recognizable lay categories and/or established research? Our analysis returned several situations where this appears to be the case. Trying to answer this question often involved examining demographics.

#### 4.3.1 Singlehood and gender

Gender distribution across profiles revealed notable trends. Men were over-represented in casual-dater profiles—despite making-up only 42.5% of the full sample, they comprised 57.0% of the Happy Explorers, 53.3% of the High-Social Explorers, and 55.2% of the Stressed Explorers. In contrast, women (57.5% of the full sample) were over-represented in non-dater profiles, comprising 67.6% of Happy Non-daters, 71.2% of Introverted Non-daters, and 69.8% of Distressed Non-daters.

Partner-seeker profiles varied by perceived pressure to partner. Relaxed Seekers (low-pressure partner-seekers) had slightly more men (50.6%), while Complex Seekers (high-pressure partner-seekers) had more women (64.2%). This aligns with research showing women seeking partners tend to report higher pressure (Girme et al., 2023). Among those not seeking partners, women favored non-dater profiles, whereas men preferred casual-dater profiles.

These trends reflect prior findings that single women report greater satisfaction with singlehood, sex, and life, plus a lower desire to partner, compared to single men (Hoan and MacDonald, 2024). This may help explain the gender split: women in non-dater profiles and men in casual-dater profiles likely reflect differing sources of fulfillment in singlehood.

#### 4.3.2 Age

Age tended to covary with perceived pressure to partner: older singles were over-represented in low-pressure profiles, while younger singles were over-represented in high-pressure profiles. This pattern appeared to vary according to romantic goals. Among 45- to 65-year-olds, more belonged to the Happy Non-daters (77.6%), Happy Explorers, (65.4%), and Relaxed Seekers (55.6%)—all low-pressure profiles. Among, 18- to 24-year-olds, more belonged to the Distressed Non-daters (42.0%), High Social Explorers (33.1%), and Lonesome Seekers (35.2%)—all high- or medium- pressure profiles.

#### 4.3.3 Recognizable profiles

Several profiles do appear to correspond to real-world, identifiable groups of singles. Complex Seekers (mostly socially connected, high-pressure women dissatisfied with singlehood) seem to mirror the “always the bridesmaid, never the bride” stereotype. The typical individual in this profile appears to be a woman of marriageable age who strongly desires a partner but has not yet found one, resulting in considerable anxiety. Despite the cultural prominence of this narrative, this profile comprises only 7.9% of the full sample—reflecting a small minority.

Stressed Explorers also stand out. More likely to be high-pressure men with large incomes and a child at home, they report both high life satisfaction and high ill-being. It is easy to imagine the modal person in this profile as the stereotypical “alpha male”—a competitive, high-stress, high-reward person with an experimental romantic history (e.g., polyamory, friends with benefits) and strong desire to find a committed partner.

However, our most noteworthy (and happiest) profiles are the Happy Explorers and Happy Non-daters. Both groups are older and more likely divorced, though at least half never married. Happy Explorers skew male (57.0%) and comprise 8.3% of the sample; Happy Non-daters skew female (67.6%) and make up 22.0%.

A special note is merited for Happy Non-daters, who resemble the colloquial “cat ladies” stereotype—a disparaging label most famously used by Republican Vice Presidential candidate J. D. Vance, who directed it as an insult toward the Democratic Presidential candidate, Kamala Harris, during the 2024 presidential race. Often older, Happy Non-daters report high well-being and self-esteem with low ill-being and a strong preference for solitude. Although they are dissatisfied with their friendships, they are satisfied with their family relationships. Historically, women have been shamed for remaining single and told they will become miserable with age. Contrary to this notion, our Happy Non-daters are thriving. Prior research supports this: as pressure to couple declines with age, many singles grow more confident and satisfied (DePaulo, 2023c). Many build rich social networks and embrace solitude as fulfilling, not lacking.

On that note, one of the most poignant findings in our data is actually a non-finding. Our data (despite being capped at age 65) show no “old and miserable” profile. While there are certainly some people who are older and less happy, there is no corresponding profile that suggests “old and miserable” is a common outcome for singles.

### 4.4 Limitations

Our study has several limitations to consider when generalizing our findings. First, as this study was not pre-registered and used an exploratory approach, replication is needed to confirm and extend the results. Data were collected in the latter half of the COVID-19 pandemic, before the Omicron variant. Thus, pandemic-related factors may have influenced group means, particularly among casual-daters. Park et al. (2023) found that psychological motives, such as self-protection and disease avoidance, shifted between pre- and post-lockdown phases, varying by profile. Replicating our analyses with a post-pandemic sample may clarify how these patterns evolve across contexts.

Our study was also a secondary analysis, constraining our choice of variables. Therefore, we could not perfectly operationalize some constructs and relied on proxies (e.g., perceived pressure to partner as a proxy for stigma). Some variables showed ceiling effects. For example, the number of married people in one’s social network was capped at “3+,” and most participants (61.8%) chose that option. Future studies could address these limitations and expand the range of intrinsic and extrinsic indicators. Additionally, the cross-sectional nature of our data precludes causal conclusions about profile membership, outcomes, and covariates.

Methodological limitations of LPA also warrant caution. As noted by Bauer and Curran (2003) and Masyn (2013), LPA can identify profiles due to non-normality in variables rather than true subgroups. While our profile solution was theoretically grounded and interpretable, this limitation may affect the robustness of our findings. Moreover, self-report measures also introduce potential biases, including social desirability and other forms of response bias. Multi-method and/or longitudinal study designs could help mitigate these concerns in the future.

Finally, our sample was constrained by age and culture. It included only individuals aged 18 to 65, offering limited insight about singles age 65+. It was also exclusively American, and thus subject to Western cultural biases (e.g., WEIRD standards; Henrich et al., 2010). Although our profile solution may generalize to similar cultures, results may vary along Hofstede’s (2011) dimensions, such as Individualism/Collectivism and/or Masculinity/Femininity.

### 4.5 Future directions and implications

In terms of future directions, an important next step is to replicate our findings. A less resource-intensive approach could use variable-centric analyses. For example, in our LPA profile solution, casual-dater and non-dater profiles show similar male-to-female ratios regardless of perceived pressure. However, partner-seeker profiles show pressure-related variation in their gender ratio. Replicating this pattern via regression analysis with a modest-sized sample may provide convergent evidence for our profile solution.

#### 4.5.1 Implications for research

In terms of practical implications for research, we see three takeaways. First, our profile solution may contribute to a typology of singlehood. This study suggests that data-driven methods can meaningfully group singles into types and the resulting profiles somewhat resemble lay categories of singles. A practical next step is to study heterogeneity holistically by examining data-derived profiles (e.g., LPA solutions such as Girme et al., 2023, Pepping et al., 2024, and the present study), naturalistic categories (e.g., incels, single parents, elder singles) and theory-based classifications (e.g., Stein’s voluntariness and stability, Kislev’s identity categories) to assess the overlap across approaches.

Second, values (e.g., autonomy, connectedness, exploration) may underlie our romantic goals and perceived pressure framework. Constructs like preference for solitude, perceived pressure to partner, and romantic goals may reflect deeper value orientations. Directly measuring singles’ values in the future may offer greater insight. For example, Girme et al. (2023) found that autonomy-related motives are key to happiness in singlehood. Future research could explore how values like autonomy interact with romantic goals and perceived pressure to partner.

Third, our findings address a long-standing tension in singlehood research. Before DePaulo and Morris (2005), studies focused on how singles could find a romantic partner, assuming most singles wanted one. Work after the early 2000s shifted towards studying singles’ well-being independent of partner seeking. Clearly, however, finding a partner remains important for many singles—nearly half of our sample (45.8%) sought long-term partners. Singlehood research should adopt a dual approach—examining both partner-seeking and non-partner-seeking—since neither group is large enough to warrant exclusive focus.

This insight implies that treating singles as an undifferentiated group is likely counterproductive. We recommend distinguishing between different types of singles since combining these groups may repress or dilute relevant effects. Dividing singles into the three romantic goal categories used here may offer greater clarity. For example, a researcher examining the link between preference for solitude and well-being may find weak or inconsistent results when focusing on casual-daters and partner-seekers, who tend to report lower preference for solitude. However, by focusing on non-daters, the same researcher may uncover stronger and more reliable associations between preference for solitude and well-being.

#### 4.5.2 Clinical and practical implications

In terms of clinical and practical applications, we see two potential takeaways.

First, our findings highlight the value of using romantic goals to understand the links between singlehood and well-being. We identified three profiles: casual-daters, who tend to be happier; partner-seekers, who tend to be less happy; and non-daters, whose well-being is closely tied to external pressure. While these findings require replication, they suggest that exploring romantic goals and values could be a productive starting point in clinical work with singles. Romantic goals may also serve as leverage for change; for instance, if a patient is unhappy due to their unfulfilled partner goals, a therapist might explore whether adjusting those goals could enhance well-being. This aligns with Hostetler’s (2009) work on secondary control strategies.

Second, our findings may inform public discourse on singlehood and age. Although future research is needed to examine older adults (65+) and compare them to married peers, our data suggest that later-life singlehood is not inherently unhappy. In fact, the least happiest singles are often younger—those facing the greatest pressure to find a partner and have children—who report more stress, anxiety, and dissatisfaction. By contrast, many older singles appear to thrive. Despite disparaging labels like “cat ladies” or “mama’s boys” (Dupuis and Girme, 2024), our profile solution suggests a substantial portion do quite well—though longitudinal studies are needed to confirm these findings. Together, these insights may help challenge cultural narratives that older singlehood inevitably leads to misery.

## 5 Conclusion

So, who does singlehood best? By the measures we have employed in this study the answer seems fairly clear—older singles who are not seeking a partner and experience low perceived pressure have higher well-being and lower ill-being than other singles. Jointly we have referred to these groups as possibly “single at heart,” and we think their existence (and the absence of a profile of older miserable singles) should contribute to changing the public discourse on singlehood. However, other groups also merit mention—younger, successful, casual dating singles with high levels of both well-being and ill-being; unhappy younger singles who experience high perceived pressure but are well-satisfied with singlehood.

Our research, then, suggests the answer is complex. Some types of singles are happier than others, but our results suggest singles’ wellness exists within the context of their romantic goals and the pressure created by those around them. This suggests a simple path forward for those who seek to understand how to deal with the single population; listen to them, respect their self-determination, and create a societal context where they can be themselves without stigma. We are already well on the way to creating a society where this is the case, and the growing number of singles across the world highlights the importance of continuing to do so.

## Data Availability

The datasets presented in this article are not readily available as the participants have not consented to share an open/public dataset. Requests to access these datasets should be directed to lisawalsh08@gmail.com.
